# The earliest evidence for modern-style plate tectonics recorded by HP–LT metamorphism in the Paleoproterozoic of the Democratic Republic of the Congo

**DOI:** 10.1038/s41598-018-33823-y

**Published:** 2018-10-18

**Authors:** Camille François, Vinciane Debaille, Jean-Louis Paquette, Daniel Baudet, Emmanuelle J. Javaux

**Affiliations:** 1 Early Life Traces & Evolution-Astrobiology, Department of Geology, B18, University of Liège, 4000 Liège, Belgium; 2 Laboratoire G-Time, Université Libre de Bruxelles, CP 160/02, 50 Avenue F.D. Roosevelt, 1050 Brussels, Belgium; 3 Université Clermont Auvergne, CNRS, IRD, OPGC, Laboratoire Magmas et Volcans, F-63000 Clermont-Ferrand, France; 4 Earth Sciences Department, Royal Museum for Central Africa, Tervuren, Belgium

## Abstract

Knowing which geodynamic regimes characterised the early Earth is a fundamental question. This implies to determine when and how modern plate tectonics began. Today, the tectonic regime is dominated by mobile-lid tectonics including deep and cold subduction. However, in the early Earth (4.5 to 2 Ga) stagnant-lid tectonics may also have occurred. The study of high pressure–low temperature (HP–LT) metamorphic rocks is important, because these rocks are only produced in present-day subduction settings. Here, we characterize the oldest known HP–LT eclogite worldwide (2089 ± 13 Ma; 17–23 kbar/500–550 °C), discovered in the Democratic Republic of the Congo. We provide evidence that the mafic protolith of the eclogite formed at 2216 ± 26 Ma in a rift-type basin, and was then subducted to mantle depths (>55 km) before being exhumed during a complete Wilson cycle lasting ca. 130 Ma. Our results indicate the operation of modern mobile-lid plate tectonics at 2.2–2.1 Ga.

## Introduction

**Figure 1:**
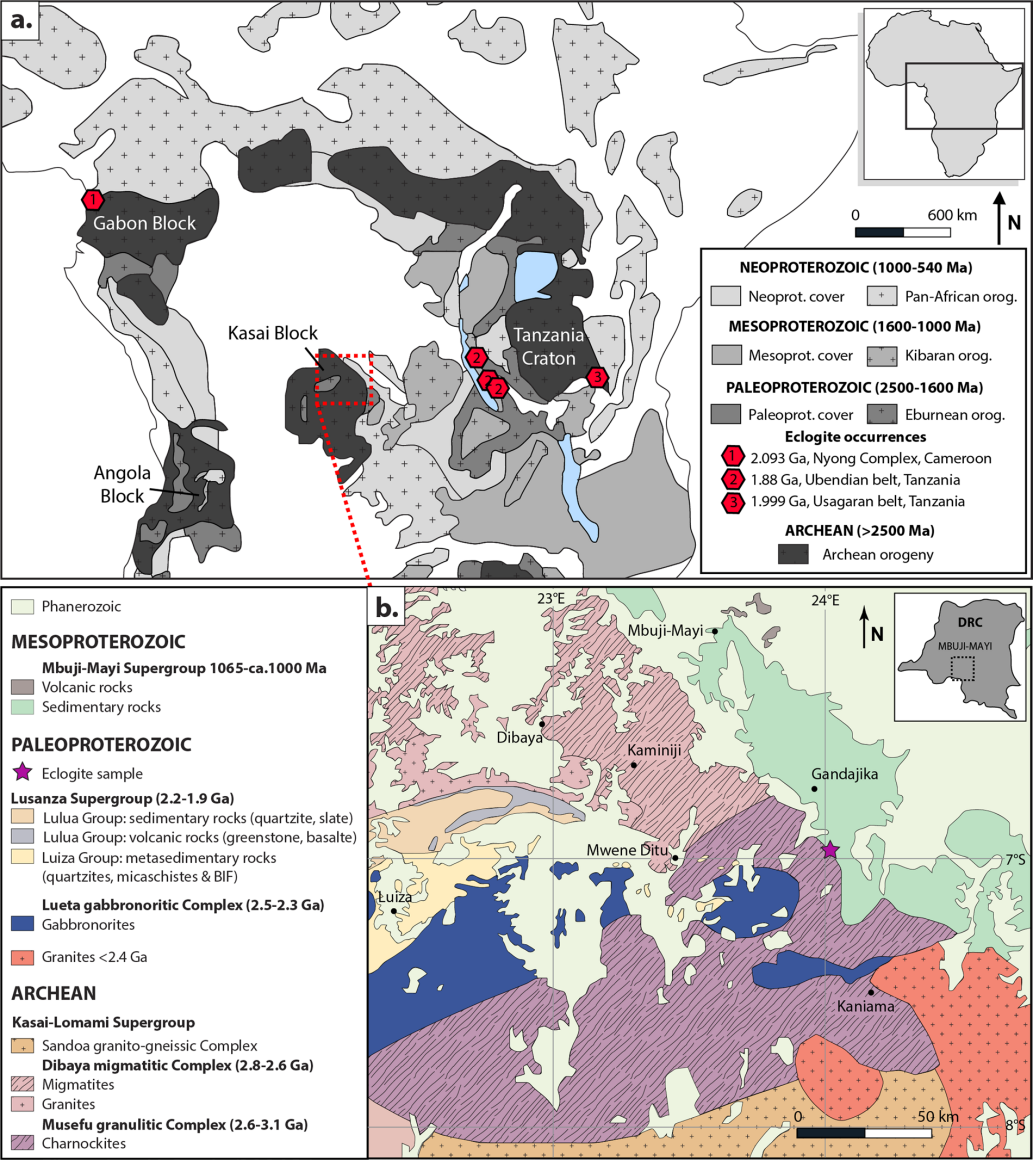
Simplified geological map of Central Africa with occurrences of Paleoproterozoic eclogites (**a**) and magnification of the geological map for the studied area (**b**). Eclogite age data from^7–10^. Maps modified after^17^, the 1:200 000 geological maps of Geological Survey of DRC (Sheets: S7/23, S7/22, S8/23 and S8/22) and the Commission for the Geological Map of the World CGMW map.

Eclogites are high-pressure metamorphic rocks mainly composed of omphacite and garnet. Their pressure–temperature conditions of formation are characteristic of modern subduction zones and, as such, they have been considered as representative of subduction processes in the geological record^1,2^. Few occurrences of true eclogites with precise ages have been described from the Archean to Paleoproterozoic rock record, thus the pattern and timing of early Earth tectonics are still heavily debated. The oldest currently proposed eclogites from an orogenic belt are recorded from the Belomorian Belt in Russia, which are dated between 1.9 and 2.8 Ga^3–5^. Other relicts of eclogites are found in Paleoproterozoic orogens, in the 1.9 Ga Snowbird zone from the Canadian Shield^6^ and in the 1.9–2.0 Ga Ubendian-Usagaran Belt of Tanzania^7–9^ (Fig. 1a). The oldest known high temperature eclogites (18–20 kbar and 800 °C; 2093 ± 45 Ma) with a MORB-like chemistry occur at the northwestern margin of the Congo Craton in the Nyong Complex of Cameroon^10^ (Fig. 1a).

The apparent geothermal gradient recorded by rocks may be used to discriminate geodynamical processes in the Early Earth, and more particularly to infer whether or not deep and cold subduction, i.e. ‘modern-style’ plate tectonics was in operation^11,12^. However, relying only on the apparent geothermal gradient might be misleading. Indeed, Archean metamorphic rocks not only record high apparent geothermal gradients, but also a large range of other possible apparent geothermal gradients (i.e. 15–30 °C/km), including low values similar to modern subduction^13^. The maximum pressure attained will be limited in the case of sagduction, which is a partial convective overturn due to density contrast between dense (ultra)mafic covers into their granitoid crustal basement coupled to partial melting in the lower crust^13^. As such, it is an intracrustal process and the maximum pressure recorded depends on the crustal thickness (greenstones and crustal basement^13–16^). Thus, a very high pressure (i.e. >15–20 kbar ≈ 50–65 km) seems difficult to reconcile in the case of stagnant-lid and sagduction tectonics. Therefore, the study of (U)HP-LT rocks, including eclogites, seems to be a robust tool to evidence modern-style (deep and cold subduction) tectonics. Here, we focus our study on eclogites discovered in Democratic Republic of the Congo (DRC) in the Archean to Paleoproterozoic Congo Craton.

## Sample Description

**Figure 2:**
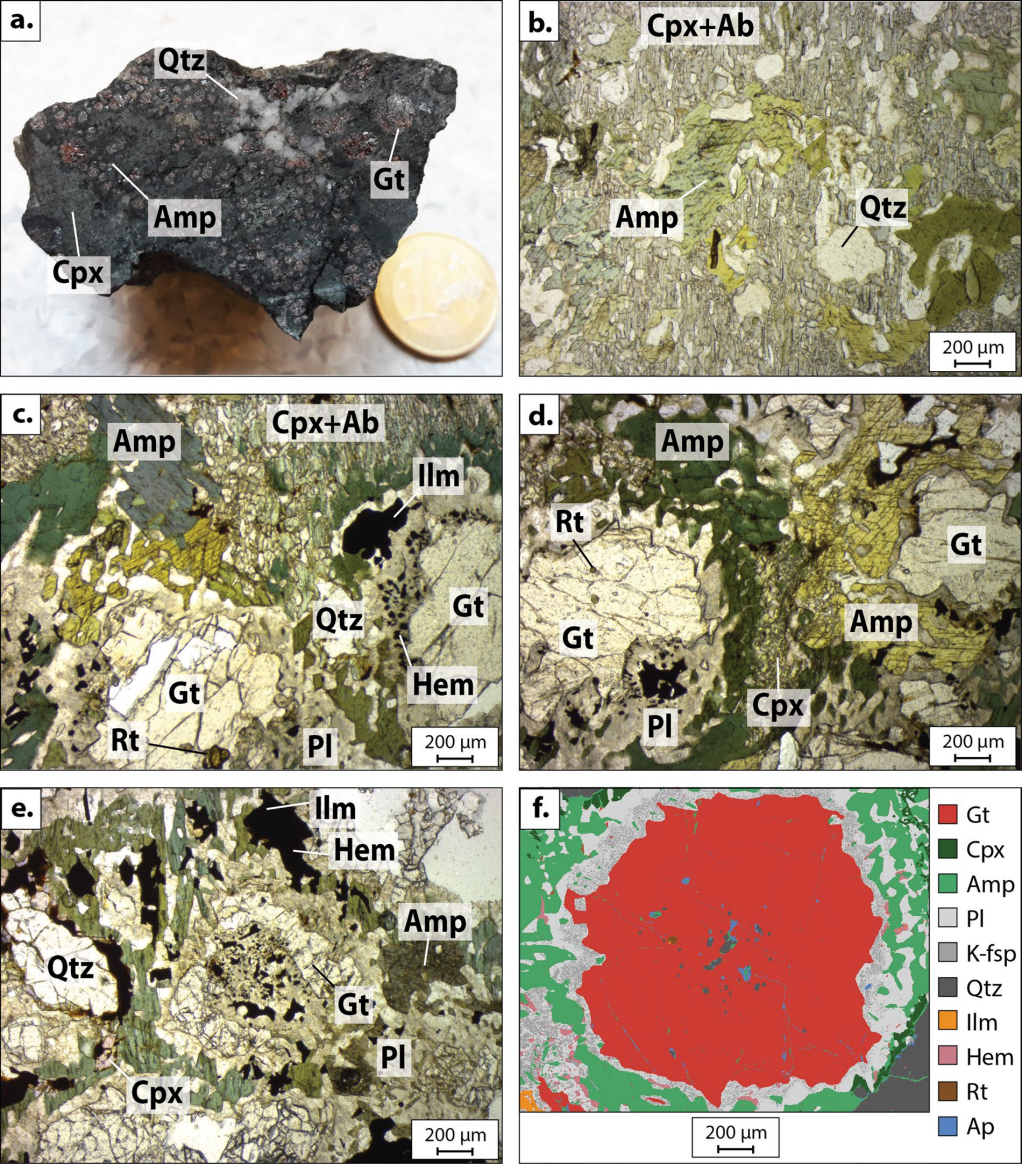
Main minerals contained in the studied eclogite. (**a**) Photograph of the sample. (**b**) Microphotographs of clinopyroxene and plagioclase matrix containing amphibole and quartz crystals, (**c,d**) symplectites of feldspar and iron-oxides around garnet in a clinopyroxene, plagioclase, amphibole and quartz matrix. Garnets present rutile inclusions, and sometimes (**e**) an atoll-shaped. (**f**) Mineral mapping of a garnet containing rutile, quartz and apatite inclusions in an amphibole, feldspar, clinopyroxene and quartz matrix. Feldspar close to the garnet are K-rich. Mineral abbreviations from^53^.

The studied sample is conserved at the Royal Museum for Central Africa, Tervuren, Belgium (RG-45977 serial number collection, Fig. 2a) and was collected in 1946 by Pierre Schnock. This rock comes from the South East of Gandajika town (value in decimal degrees: S6.5-S7/E23.9–24.5 close to Kayemba Ngombe town), in the northern part of the Archean to Paleoproterozoic Kasai Block (Fig. 1) within the Congo Craton. The northern part of the Kasai Block is composed of the Musefu Granulitic Complex (2.6–3.1 Ga^17^; Fig. 1b) and the Dibaya migmatitic Complex (2.6–2.8 Ga^17,18^). This Archean Block was marked by the Eburnian–Transamazonian (2.2–1.98 Ga) orogeny, which resulted from the accretion of the Congo Craton and the Brazilian São Francisco Craton^19^. The associated metamorphism has been dated at 2.05 Ga in Cameroon^10,20^ (Fig. 1a) and at 2.10–2.07 Ga in Brazil^21^. The area records the emplacement of the Lueta gabbronoritic Complex and the Lusanza Supergroup (2.2–1.9 Ga^17,19^) during the Paleoproterozoic. Some enclaves of the upper Lusanza Supergroup have been described within the Musefu granulitic Complex close to Mwene Ditu town (Fig. 1b). No evidence of HP rocks (blueschist or eclogite) has previously been described from the area, other than the ca. 70 Ma^22^ eclogite xenoliths from kimberlites found close to Mbuji-Mayi town.

The chemical composition of the eclogite is basaltic (Table 1 and see Supplementary Fig. S1) with SiO_2_ = 51.6 wt.%, Na_2_O = 2.7 wt.% and TiO_2_ = 1.2 wt.%. It contains low K_2_O (<0.5 wt.%), but high CaO (11.3 wt.%). A primitive mantle-normalised trace element diagram (see Supplementary Fig. S2) shows an enriched-MORB signature.

**Table 1:** Whole rock composition and trace elements for our eclogite sample and for eclogite sample from Cameroon^10^.

Sample	RG45977	157-1	158-1	159b-1	161b-1
SiO2	51,6	48,92	49,97	50,69	46,56
Al2O3	12,5	13,62	13,93	13,86	13,56
TiO2	1,2	0,8	1,01	0,52	1,23
Fe2O3	12,7	13,71	13,29	12	16,48
MnO	0,2	0,23	0,2	0,27	0,21
MgO	7,8	7,47	7,84	9,05	8,23
CaO	11,3	12,73	11,95	10,94	12,13
Na2O	2,7	2,03	1,68	1,92	1,75
K2O	0,5	0,11	0,03	0,22	0,09
P2O5	0,1	0,07	0,08	0,05	0,14
**Total**	**101,0**	**99,69**	**99,98**	**99,52**	**100,38**
LOI	0,4	—	—	—	—
Rb	15,38	1,16	0,69	1,65	0,50
Sr	204,88	41,10	64,90	41,60	64,60
Y	18,46	22,60	21,60	19,00	36,10
Zr	73,53	39,30	56,60	35,20	91,20
Nb	10,91	1,92	2,65	1,58	3,93
Ba	87,58	16,50	6,83	26,30	5,44
La	8,98	1,10	3,05	1,95	4,24
Ce	19,73	3,35	8,18	5,47	12,30
Pr	2,63	0,59	1,31	0,92	2,03
Nd	12,24	3,31	6,66	4,67	10,40
Sm	2,93	1,43	2,22	1,51	3,49
Eu	0,87	0,58	0,79	0,56	1,16
Gd	3,47	2,37	2,77	1,98	4,50
Tb	0,69	0,51	0,53	0,40	0,85
Dy	3,23	3,45	3,39	2,75	5,53
Ho	0,79	0,75	0,72	0,62	1,18
Er	2,32	2,16	2,06	1,86	3,47
Tm	0,34	0,32	0,30	0,28	0,51
Yb	2,11	2,08	1,93	1,82	3,28
Lu	0,32	0,31	0,29	0,28	0,49
Hf	2,26	1,10	1,44	0,98	2,20
Ta	0,83	0,10	0,16	0,10	0,23
Pb	5,60	0,71	0,41	0,44	1,88
Th	1,32	0,03	0,36	0,11	0,14
U	0,24	0,05	0,07	0,04	0,07
Cr	91,25	258,00	144,00	511,00	76,60
Co	46,09	73,90	56,40	61,40	69,60
Ni	64,06	267,00	146,00	215,00	122,00
Cu	25,25	175,00	191,00	9,51	68,20
Zn	80,65	112,00	102,00	90,80	102,00
Ga	16,31	16,40	17,20	14,60	17,90

The sample is a retrogressed eclogite and consists mainly of garnet, clinopyroxene, amphibole, rutile, feldspar, ilmenite, hematite, quartz and pyrite (Fig. 2, see Supplementary Table S1 and Fig. S3). Garnets are a solid solution between almandine (53–60%), grossular (21–29%), pyrope (11–19%) and spessartine (1–7%; see Supplementary Table S1 and Fig. S4a**)**. They present a zoning pattern with a Fe- and Mn-rich core, and Ca- and Mg-rich rims. Rutile, clinopyroxene, amphibole and quartz are present in inclusions in garnet (Fig. 2f) and form the first paragenesis. No coesite was found. Corona textures around garnet are retrograde (Fig. 2c–f). Some garnets display atoll-shaped microstructures (Fig. 2e). A similar garnet shape was observed in other eclogitic rocks^23–25^. Clinopyroxenes have a pale greenish colour and constitute the major part of the matrix, often associated with albite-rich plagioclases in symplectites, which grew during the decompression (Fig. 2b). They are Ca- and Na-rich (see Supplementary Table S1) and have a composition of aegirine-augite. The XMg content varies from 0.016 to 0.08, the XCa between 0.88 to 0.98 and the XFe between 0.05 and 0.40. The jadeite amount (XJadeite) is between 2.0 and 4.0. However, as this eclogite is retrogressed, the initial composition of clinopyroxene was close to omphacite. The composition of omphacite was estimated by adding the oxide wt.% of clinopyroxene and the oxide wt.% of albite-rich plagioclase (SiO_2 (Cpx)_ + SiO_2 (Pl)_; TiO_2 (Cpx)_ + TiO_2 (Pl)_; …) analysed by electron microprobe (see Supplementary Table S1). The estimated compositions of XJadeite in omphacite were close to 24–28 wt.% and probably below 30 wt.%. Amphiboles have an intense greenish colour. They are mainly calcic, with Mg content ranging between 0.5 and 0.65, the Na + K content <0.05 and the Si content <7.1 (hornblende: pargasite to ferro-edenite; see Supplementary Table S1 and Fig. S4b). They occurred between garnet and pyroxene and sometimes within garnet (Fig. 2c,d). Feldspars are rich in Na and Ca when close to clinopyroxene and amphibole, and richer in K close to garnet (Fig. 2f). Rutile occurs in the matrix and mainly as inclusions within garnet (Fig. 2c,d). Ilmenite, hematite and titanite commonly replace rutile in the matrix (Fig. 2c,e). Apatite and zircon occur as accessory minerals. Kyanite is absent and, except amphibole, no hydrated mineral is present.

## Thermobarometry

**Figure 3:**
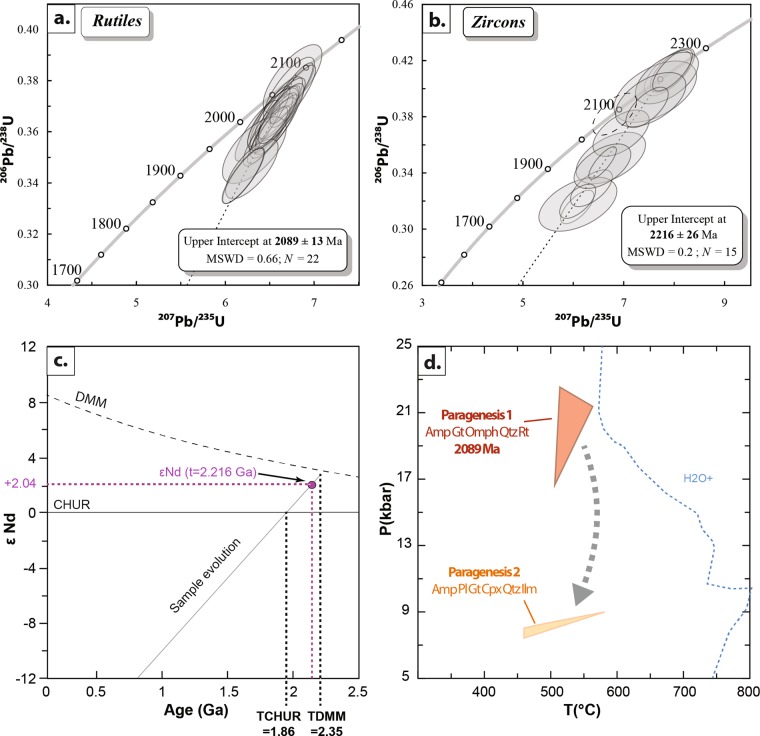
Dating, Nd-epsilon and thermobarometry estimates for the sample. (**a**) Concordia diagrams for LA-ICP-MS U–Th–Pb analyses on 18 rutiles (22 analyses) and (**b**) on 15 zircons (dotted ellipse represents analyse of the metamorphic zircon). Error ellipses are 2σ.(**c**) Nd-epsilon *vs* age diagram for the eclogite. Model of Depleted Mantle is from^39^ (**d**) P-T-t path of the eclogite sample. Colored boxes correspond to the first paragenesis (Gt-Omph-Amp-Qtz-Rt) and to the second paragenesis (Gt-Cpx-Amp-Pl-Qtz-Ilm) in Perple_X pseudosection. Mineral abbreviations from^53^.

In order to constrain the P–T conditions, we performed thermodynamic modelling (see Supplementary Fig. S5) using the phase-diagram calculation software Perple_X^26^ (version 6.8.3) and the self-consistent thermodynamic database and mineral solution models (solution_model_682; upgrade 2018). Bulk-rock compositions were calculated in the TiMnNaCaFMASH system from modal phase proportions. Mineral solution models used are Grt(WPH)^27^, Opx(HP)^28^, Cpx(HP)^28^, Omph(GHP)^29^, Pl(h)^30^, Chl(HP)^31^ and cAmph(DP)^32^. Water content was estimated at 0.4 wt.% using a xH_2_O *vs*. temperature diagram (at 17 kbar). Considering the pseudosection, the first paragenesis of garnet, omphacite, amphibole, quartz and rutile is stable over a large range of P–T between 400 and 550 °C for a pressure exceeding 10 kbar but lower than 24 kbar because no coesite was present. Adding the isopleths modelled for this assemblage for garnet: XPyrope (11–15 wt.%) and for XJadeite in the estimated omphacite (<30 wt.%), P–T conditions for the first paragenesis are estimated between 17 to 23 ± 1 kbar and 500–550 ± 50 °C (Fig. 3d and Supplementary Fig. S5, see^33,34^ for the associated errors). The exhumation is characterized by higher content of XPyrope (15–19 wt.%), a low content of XJadeite in the clinopyroxene (2–4 wt.%), the appearance of plagioclase by the substitution of omphacite in augite and albite (XAlbite: 61–81 wt.%) and the appearance of ilmenite and hematite (Fig. 3d and Supplementary Fig. S5) around 7.5–9.5 ± 1 kbar and 450–575 ± 50 °C.

## U-Pb Dating

Zircons (20–100 µm) show subhedral to oval shapes, some grains displaying irregular and poorly visible zoning with locally preserved thin overgrowths (see Supplementary Fig. S6). Their morphology is very similar to that of zircons observed in some Variscan eclogite-facies meta-gabbros^35^. The rutiles (50–500 µm) generally appear homogeneous in BSE and reflected light images (see Supplementary Fig. S7). U-Pb ages were determined by Laser Ablation Inductively Coupled Plasma Mass Spectrometry (LA-ICP-MS) at the Laboratoire Magmas et Volcans, Clermont-Ferrand, France (Fig. 3a,b and Table 2). All the dated rutile grains contain a low U content of about 20 ppm and no Th, as often observed in rutiles^36^. A total of 22 spots was performed on 18 rutile crystals (see Supplementary Fig. S7) and yields a discordia line with an upper intercept at 2089 ± 13 Ma (MSWD 0.66, Fig. 3a), the lower intercept being at the origin of the concordia diagram within uncertainties. However, diffusion-induced resetting is unresolvable in LT eclogites^36^ and rutiles in eclogites provide similar ages than eclogitic zircons^37^. Consequently, the obtained 2089 Ma upper intercept can be confidently interpreted as the age of the eclogite-facies event. These results evidence the highest pressure so far reported for eclogitic facies metamorphism in the Paleoproterozoic. Fifteen zircons crystals were analysed (sorted and in thin section; see Supplementary Fig. S6) and yielded a discordia line with an upper intercept age of 2216 ± 26 Ma (MSWD 0.2, Fig. 3b), the lower intercept being, as for rutiles, at the origin of the diagram within uncertainties. These sub-euhedral zircons display a weak and irregular zoning and a mean Th/U ratio of 0.4 regardless of the variable amounts of U, rather favouring a magmatic protolith^38^. A single zircon grain is concordant at 2087 ± 45 Ma (Table 2) and is characterized by a lower Th/U ratio of 0.05. It provides a similar age than the rutiles and may have been dissolved and recrystallized during the eclogite facies event. On the contrary, all the other zircons are interpreted as dating the magmatic stage of the mafic protolith at 2216 Ma.

**Table 2:** Rutile and zircon U-Th-Pb data obtained by *in situ* Laser Ablation ICP-MS.

Rutiles	*Pb*	*Th*	*U*			2 σ absolute		2σ absolute	error	Age (Ma)	2 σ error
analysis	ppm*	ppm*	ppm*	Th/U	^207^Pb/^235^U**	^207^Pb/^235^U	^206^Pb/^238^U**	^206^Pb/^238^U	correlation	^207^Pb/^206^Pb	^207^Pb/^206^Pb
RG-45977/Rt01	5,5		16		6,584	0,203	0,366	0,010	0,87	2125	61
RG-45977/Rt02	4,7		13		6,750	0,272	0,381	0,011	0,72	2099	76
RG-45977/Rt03	4,0		12		6,354	0,205	0,360	0,010	0,84	2089	63
RG-45977/Rt04	3,1		8,8		6,709	0,236	0,377	0,010	0,79	2108	68
RG-45977/Rt05	4,8		14		6,751	0,211	0,374	0,010	0,86	2131	61
RG-45977/Rt06	5,4		16		6,543	0,225	0,366	0,010	0,80	2115	67
RG-45977/Rt07	6,3		19		6,481	0,197	0,366	0,010	0,88	2097	60
RG-45977/Rt08	6,5		19		6,597	0,218	0,369	0,010	0,82	2112	65
RG-45977/Rt09	5,6		16		6,546	0,205	0,363	0,010	0,85	2126	62
RG-45977/Rt10	2,2		6,7		6,318	0,294	0,353	0,011	0,65	2117	88
RG-45977/Rt11	5,3		15		6,534	0,211	0,361	0,010	0,83	2133	64
RG-45977/Rt12	5,2		15		6,445	0,204	0,358	0,010	0,84	2125	63
RG-45977/Rt13	5,8		16		6,576	0,205	0,363	0,010	0,85	2138	62
RG-45977/Rt14	11		30		6,737	0,200	0,375	0,010	0,88	2121	60
RG-45977/Rt15	5,2		14		6,630	0,215	0,369	0,010	0,82	2122	64
RG-45977/Rt16	7,2		20		6,507	0,202	0,363	0,010	0,85	2117	62
RG-45977/Rt17	11		32		6,481	0,195	0,359	0,009	0,86	2132	61
RG-45977/Rt18	2,6		7,5		6,290	0,242	0,345	0,010	0,73	2146	74
RG-45977/Rt19	1,9		5,3		6,529	0,236	0,365	0,010	0,75	2115	71
RG-45977/Rt20	16		42		6,619	0,211	0,367	0,010	0,82	2128	64
RG-45977/Rt21	7,8		22		6,199	0,192	0,343	0,009	0,84	2134	62
RG-45977/Rt22	7,6		22		6,189	0,193	0,343	0,009	0,84	2132	63
**Zircons**	***Pb***	***Th***	***U***			**2** σ **absolute**		**2** σ **absolute**	**error**	**Age (Ma)**	**2** σ **error**
**analysis**	ppm*	ppm*	ppm*	**Th/U**	^**207**^ **Pb/** ^**235**^ U**	^**207**^ **Pb/** ^**235**^ **U**	^**206**^ **Pb/** ^**238**^ U**	^**206**^ **Pb/** ^**238**^ **U**	**correlation**	^**207**^ **Pb/** ^**206**^ **Pb**	^**207**^ **Pb/** ^**206**^ **Pb**
RG-45977/Zr01	13	3,2	27	0,12	7,945	0,362	0,413	0,013	0,69	2220	83
RG-45977/Zr02	20	21	55	0,38	6,054	0,272	0,315	0,010	0,69	2217	82
RG-45977/Zr03	9,6	6,4	21	0,30	7,853	0,382	0,411	0,013	0,66	2208	88
RG-45977/Zr04	3,1	1,2	7,4	0,16	7,614	0,671	0,398	0,018	0,51	2212	156
RG-45977/Zr05	2,3	2,0	5,6	0,36	6,726	0,568	0,350	0,015	0,51	2218	150
RG-45977/Zr06	4,1	4,9	11	0,43	6,070	0,633	0,317	0,016	0,48	2211	185
RG-45977/Zr07	5,8	3,9	13	0,31	7,845	0,513	0,408	0,015	0,57	2222	117
RG-45977/Zr08	8,3	6,2	21	0,30	6,929	0,457	0,368	0,014	0,56	2182	119
RG-45977/Zr09	6,4	6,0	15	0,40	7,513	0,573	0,392	0,016	0,53	2214	136
RG-45977/Zr10	9,1	11	24	0,45	6,331	0,340	0,329	0,011	0,61	2222	97
RG-45977/Zr11	4,8	9,4	12	0,76	6,552	0,462	0,345	0,013	0,54	2201	127
RG-45977/Zr12	36	31	103	0,30	6,067	0,298	0,319	0,010	0,64	2199	90
RG-45977/Zr13	15	16	34	0,48	7,451	0,344	0,386	0,012	0,66	2225	84
RG-45977/Zr14	31	71	46	1,56	7,962	0,326	0,413	0,012	0,72	2224	75
RG-45977/Zr15	10	1,1	24	0,05	6,810	0,357	0,382	0,012	0,62	2089	96
RG-45977/Zr16	153	71	420	0,17	6,563	0,225	0,347	0,010	0,80	2192	65

*Concentration uncertainty c.20%.

**Data not corrected for common-Pb.

Decay constants^52^.

## Sm-Nd Systematics

The ^147^Sm-^143^Nd systematic gives a εNd_i_ of +2.04 at 2216 Ma (Fig. 3c and see Supplementary Table S2), i.e. slightly more enriched than the evolution of the depleted MORB mantle (DMM) at ~2 Ga (e.g.^39^). The T_DMM_ model age for the sample is relatively close, at 2350 Ma (Fig. 3c). The trace element pattern also indicates a source slightly more enriched in incompatible trace elements than the DMM (Table 1 and see Supplementary Fig. S2). This slight enrichment in incompatible elements, combined to enrichment in robust and immobile elements such a Zr, Nb and Y, can be ascribed as T(transitional)-MORB rather than E(enriched)-MORB. Because T-MORB are considered characteristic of a transitional geodynamic tectonic setting between oceanic and continental lithospheres, i.e. rifting and continental breakup^40^, crustal contamination is expected and could explain the decrease of the εNd from ~+3–4 for the DMM^39^ to +2.04 as measured in the sample at 2216 Ma. Because of the crustal contamination, the model age at 2350 Ma is a maximum, the metamorphic age at 2089 Ma being the minimum. This is coherent with the age obtained on the zircon discordia line (2216 ± 26 Ma), that is thus interpreted as the true crystallization age.

## Discussion

The 2.09 Ga eclogites of the Nyong complex of Cameroon and the 2.0 Ga eclogites of the Usagaran Belt of Tanzania have a geochemical affinity to oceanic crust and are interpreted to represent the relics of subducted Paleoproterozoic oceanic crust at the margins of the Congo Craton^7,8,10^. These eclogite occurrences with MORB-like compositions in a continental setting support the hypothesis that plate tectonics operated on Earth in the Paleoproterozoic Era, apparently in a similar fashion as in the modern Earth, since production of the eclogite facies MORB requires the subduction of an old, cold and dense lithosphere (e.g.^9,41^). Moreover, the RDC eclogite presented here is the first evidence of an entire Wilson cycle in the Paleoproterozoic comprising HP-LT subduction. These eclogites derive from a mafic protolith, with a T-MORB signature, formed at 2216 ± 26 Ma in a intra-cratonic rift-type basin inside the Congo Craton, then buried at high pressure and low temperature (17–23 ± 1 kbar and 500–550 ± 50 °C) and exhumed during a cycle of ca. 130 Ma. These observations evidence a modern-style plate tectonics at 2.2–2.1 Ga. We thus show here that modern-style plate tectonics, as evidenced by cold and deep subduction (>55 km), operated at least since the Paleoproterozoic. Because it certainly took some time of a transient regime from stagnant-lid tectonic^42^ to mobile-lid subduction, this result is compatible with a major change in Earth’s tectonic regime between 2.5 and 3.0 Ga^43^. On the other hand, it is difficult to envision how mobile-lid plate tectonics could have started since ~4.5 Ga and left no older compelling imprint, even when considering the incompleteness and preservation bias of the Archean rock record.

## Methods

### U-Pb Dating

The sample was crushed for dating and rutiles and zircons were separated using standard heavy liquids and magnetic techniques. Rutiles (50–500 µm) and zircons (20–100 µm) were hand-picked and mounted in a 1 inch epoxy disc, which was polished to expose the mid-section of grains. Zircon crystals were also found *in situ* in petrographic thin section (20–50 µm), located in the matrix as well as inside garnets. The internal structures of zircons and rutiles were investigated with backscattered electron (BSE) and cathodoluminescence (CL) images at the University Pierre & Marie Curie, Paris (France).

U-Th-Pb isotope data were measured by laser ablation inductively coupled mass spectrometry (LA-ICP-MS) at LMV (Clermont-Ferrand, France). Zircons were ablated using a Resonetics Resolution M-50 equipped with a 193 nm Excimer laser system coupled to a Thermo Element XR high resolution ICP-MS. Helium carrier gas was supplemented with N_2_ prior to mixing with Ar for sensitivity enhancement^44^. The laser was operated with a repetition rate of 3 Hz, a fluence of 3.5 J/cm^2^ and spot diameters of 15 and 33 µm for zircon and rutile, respectively. The signals of ^204^Pb (+Hg), ^206^Pb, ^207^Pb, ^208^Pb, ^232^Th, and ^238^U were acquired during each analysis^45^. Background levels were measured on-peak with the laser off for ~30 seconds, followed by ~60 seconds of measurement with the laser firing and then ~30 seconds of washout time. Reduction of raw data was carried out using the GLITTER® software package of Macquarie Research Ltd^46^. Isotope ratios were corrected for laser-induced and instrumental mass fractionation via sample-standard bracketing using the GJ-1 zircon^47^ and Sugluk-4 rutile^48^ reference materials. Concentrations of U, Th, and Pb were calculated by normalization to the certified composition of GJ-1^46^ and 91500^49^. Data were not corrected for common Pb. Concordia diagrams were generated for each sample using the Isoplot/Ex v. 2.49 software of ^50^. Error ellipses for each point are shown at the 2σ level and incorporate both internal and external uncertainties. The 91500 zircon and PCA-207 rutile^48^ were analysed along with the samples, to independently monitor the external precision and accuracy of the measurements. The pooled ages for 38 analyses of 91500 and 27 analyses of PCA-S207 conducted over the course of the study were 1064.9 ± 4.5 Ma and 1862.2 ± 6.9 Ma, respectively.

### Trace elements

Around 50 mg of powdered sample was mixed with 1 g of ultrapure lithium metaborate and tetraborate (4:1). After heating at 1000 °C, the bead was re-dissolved in 50 ml of HNO3 5% plus traces of HF. After ad-hoc dilution, the sample was measured on the Agilent 7700 ICP-MS at ULB, Belgium. BHVO standard was used to ensure the precision and reproducibility of the measurements, which was better than 5% for the elements presented here.

### Sm-Nd systematics

After crushing, ~200 mg of powder have been dissolved in a mixture of ultrapure HF:HNO_3_ (1:3). After removing the supernatant, the solid residue has been re-dissolved by the same but fresh mixture in high-pressure vessels to ensure a complete dissolution of refractory phases such as zircon. The two fractions were recombined and after evaporation, HCl 6 N was added. Once the solution was clear, a small aliquot was taken and spiked with a mixed ^150^Sm-^148^Nd spike and the mixture was equilibrated 24 h on hotplate. Both unspiked and spiked aliquots were purified on a cationic resin by rinsing in 1.5 N HCl and collecting REE in 6 N HCl. Then, REE were purified from each other using home-made HDEHP resins. Both spiked and unspiked cuts have been measured on the HR-MC-ICP-MS Nu-Plasma 1 at ULB, Belgium. For the unspiked cut, the value was corrected for mass fractionation by using the ratio ^146^Nd/^144^Nd = 0.7219, and then for the accepted Rennes Nd standard value ^143^Nd/^144^Nd of 0.511963. The internal total reproducibility (n = 9) was better than 22 ppm. The measurement was replicated and values are well-within errors. For the spiked cut, mass fractionation was calculated by iterative calculation as in^51^.

## Electronic supplementary material

Supplementary dataset 1
