# Supplementary material for: The earliest evidence for modern-style plate tectonics recorded by HP–LT metamorphism in the Paleoproterozoic of the Democratic Republic of the Congo
Source: Sci Rep. 2018 Oct 18;8:15452. doi: 10.1038/s41598-018-33823-y (PMC6193989; doi:10.1038/s41598-018-33823-y)
Supplement: Supplementary file 1 — Supplementary dataset 1 [file 41598_2018_33823_MOESM1_ESM.pdf]

The earliest evidence for modern-style plate tectonics recorded by HP–LT metamorphism in the Paleoproterozoic of the Democratic Republic of the Congo

Camille François[1,*], Vinciane Debaille[2], Jean-Louis Paquette[3], Daniel Baudet[4] & Emmanuelle J. Javaux[1]

[1] *Early Life Traces & Evolution-Astrobiology, Department of Geology, B18, University of Liege, 4000 Liège, Belgium*
[2] *Laboratoire G-Time, Université Libre de Bruxelles, CP 160/02, 50 Avenue F.D. Roosevelt, 1050 Brussels, Belgium*
[3] *Université Clermont Auvergne, CNRS, IRD, OPGC, Laboratoire Magmas et Volcans, F-63000 Clermont-Ferrand, France*
[4] *Earth Sciences Department, Royal Museum for Central Africa, Tervuren, Belgium*

*Corresponding author. Tel.: +3243669411. *E-mail address*: c.francois@uliege.be (C. François).

Supplementary Information

Geological context

The Musefu granulitic Complex consists of charnockites, enderbites, granulites and leptinytes (**Fig.1b**). These rocks are partially derived from sedimentary protoliths and are produced by an intense metamorphism of an ancient gneissic crust[1]. The Dibaya migmatitic Complex is mainly composed of migmatitic gneisses with localized amphibolite zones. The geochemistry of migmatites shows a calk-alkaline affinity[2]. Calk-alkaline granites are also present in the southern part of the complex. The Lueta gabbronoritic Complex is a large mafic plutonic complex composed of gabbronorites, garnet-bearing gabbros and amphibolites. It is in contact with the Lusanza Supergroup in the North and the Archaean complexes in the South. The age of the Lueta Complex, considered Archean[3], has been questioned and is now postulated to be Paleoproterozoic between c.a. 2.5-2.3 Ga[4,5]. The Lusanza Supergroup (2.2 - 1.9 Ga[5,6]) consists of an upper unit: the Lulua volcano-sedimentary Group composed of shales and quartzites interbedded with mafic volcanics rocks (4000 to 7000 m-depth[7]) and a lower unit: the Luiza metasedimentary Group (micaceous quartzites, BIF, micaschists and metamorphosed conglomerates). This succession lies on a normal nonconformity on the Lueta gabbronoritic Complex.

Supplementary figures

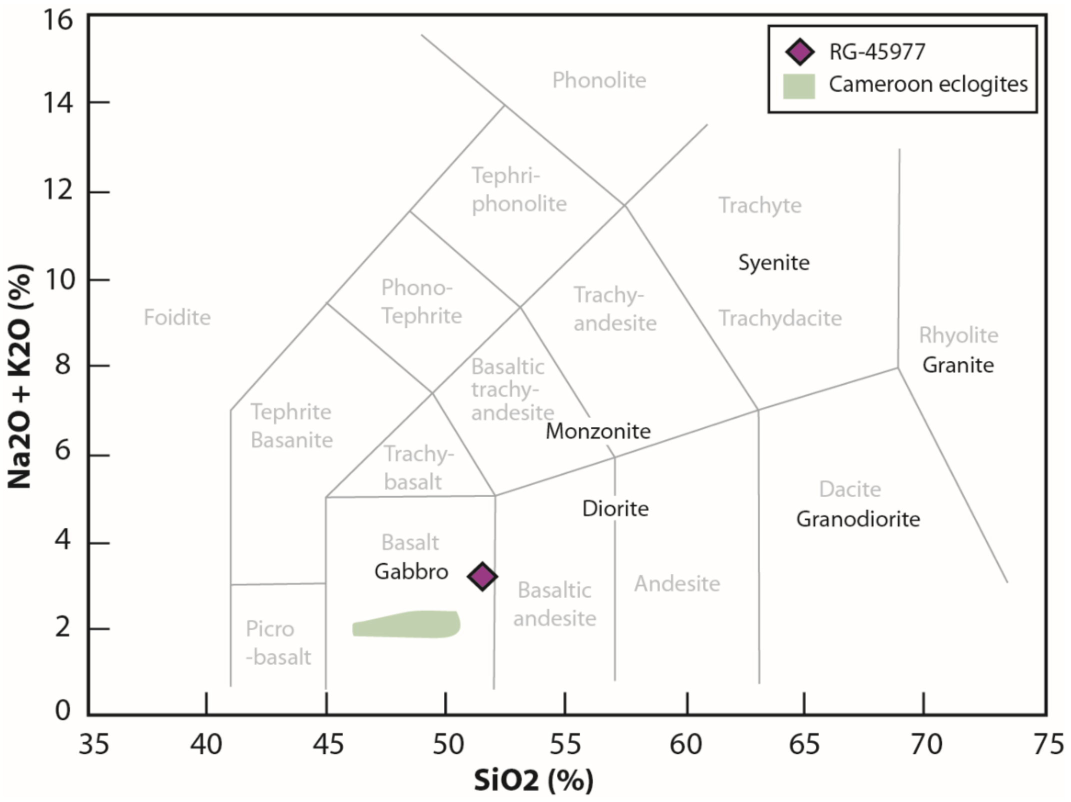

Fig.S1. $Na_2O + K_2O$ versus SiO_2 diagram of eclogite sample.

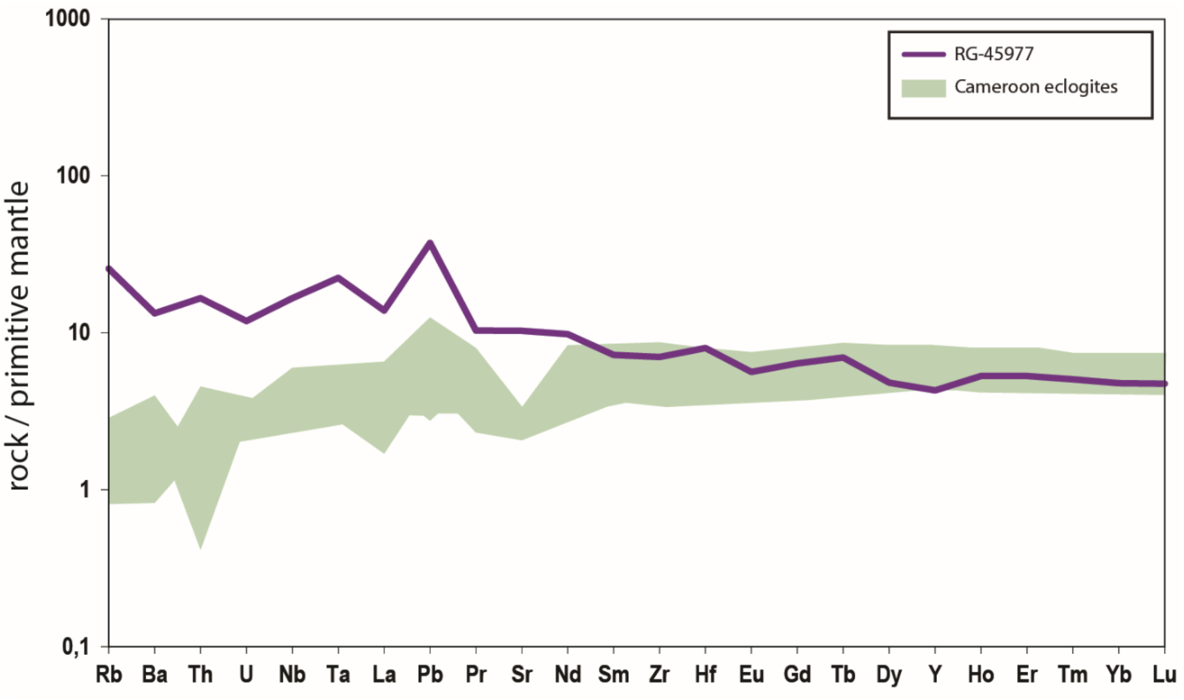

Fig.S2. Spider diagram of trace elements normalized to primitive mantle[8]. Cameroon eclogites from[10].

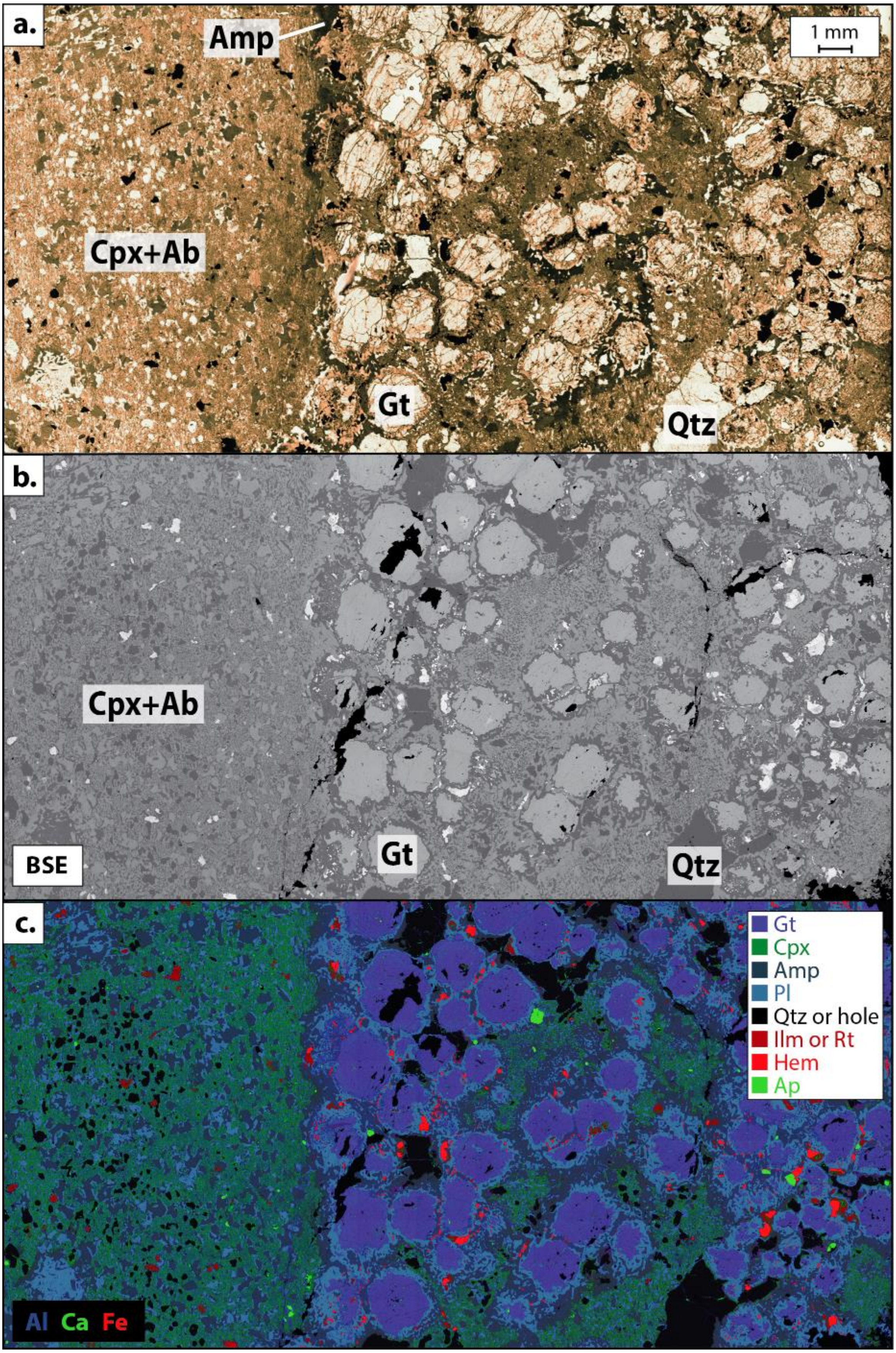

Fig.S3. (a) Mosaic of microphotographs of eclogitic sample, (b) corresponding backscattered electron (BSE) imaging and (c) combined mapping of Al, Ca and Fe.

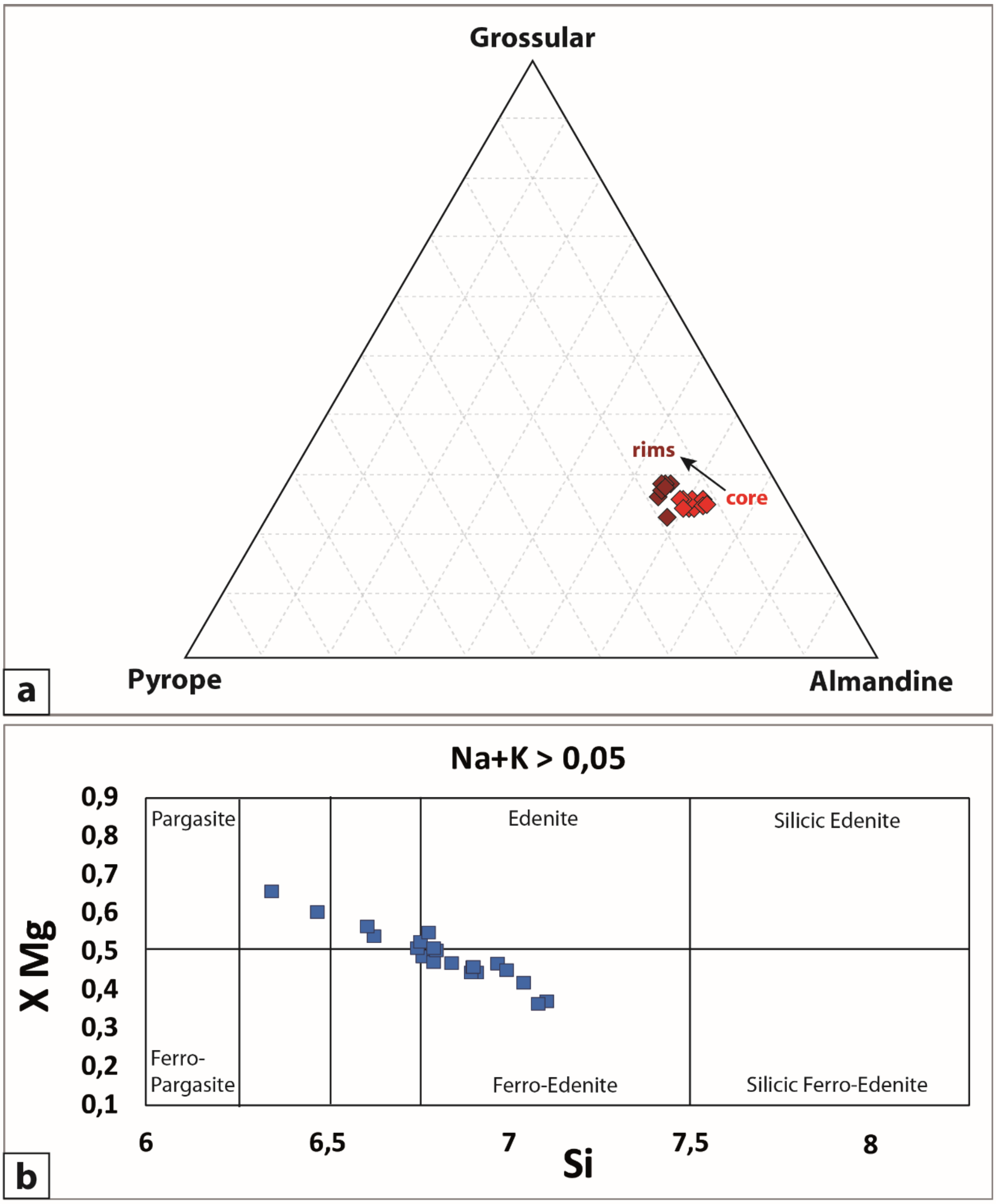

Fig.S4. Chemical composition plots of (a) garnets and (b) amphiboles.

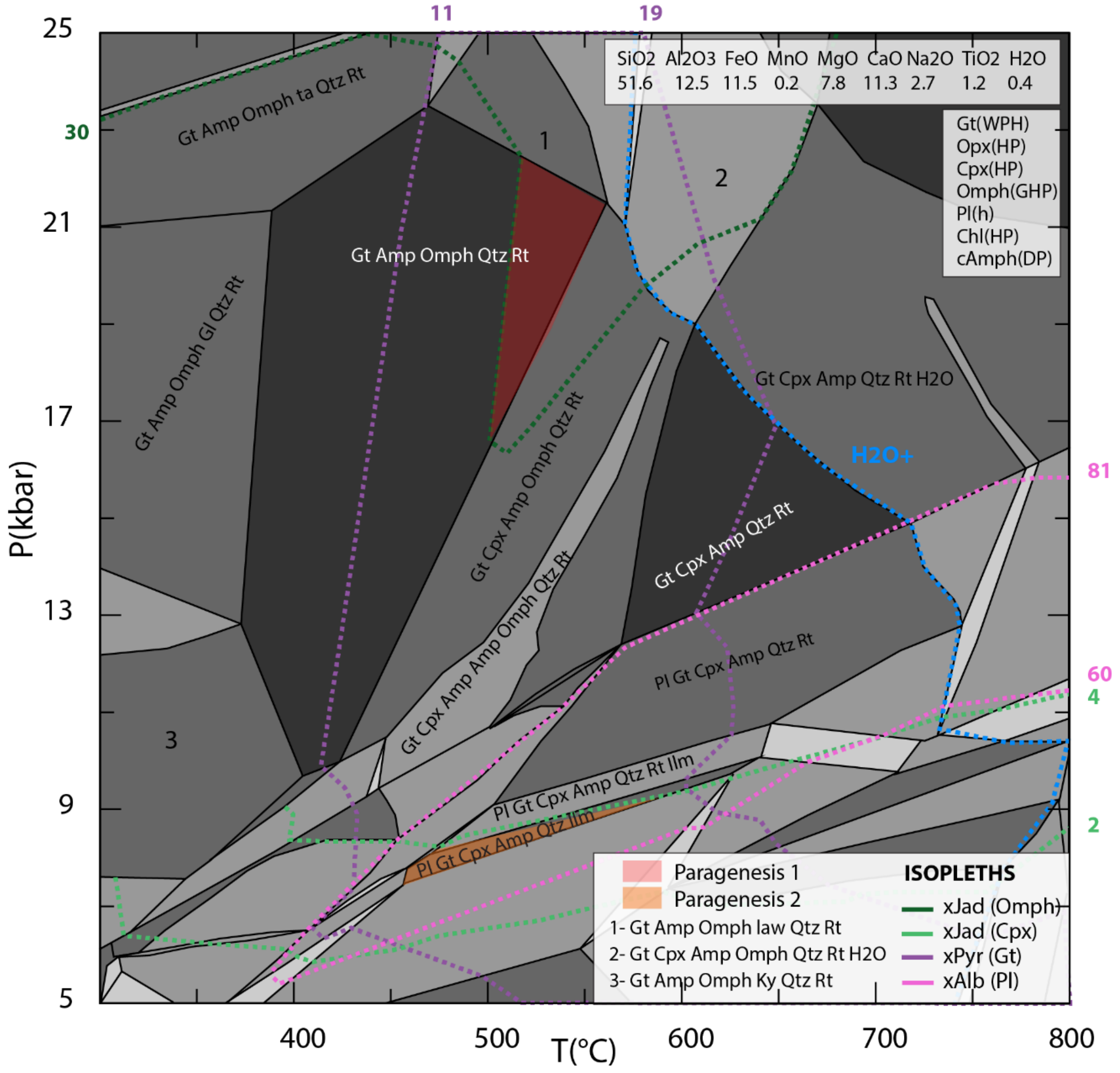

Fig.S5. Perple_X pseudosection in TiMnNaCaKFMASH system, H2O = 0.4%. Mineral abbreviations from[9]. See text for details on mineral solution models.

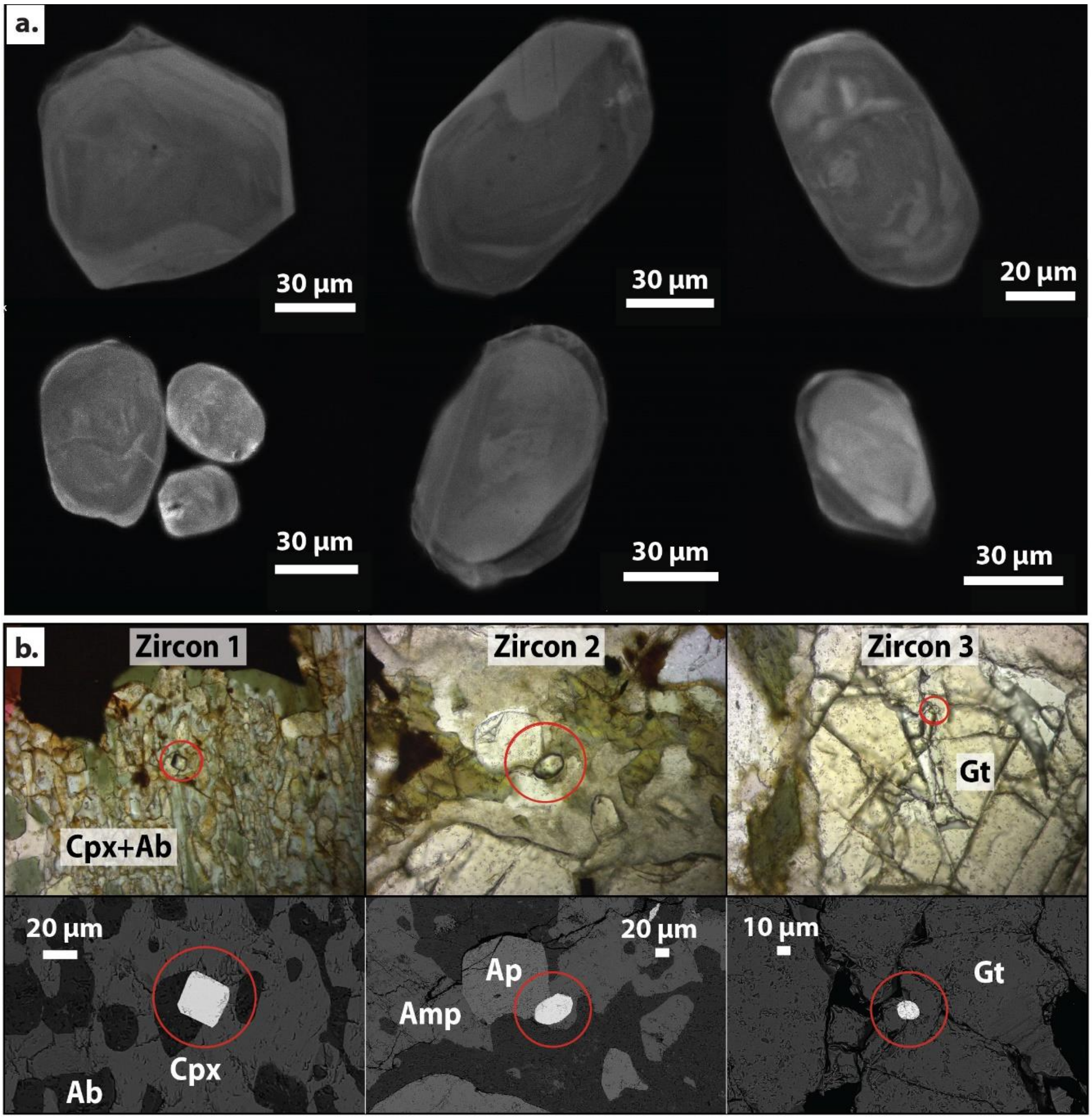

Fig.S6. Images of some studied zircons for U-Pb dating. (a) Cathodoluminescence images of sorted zircons. (b) Microphotographs (transmitted light) and backscattered imaging for three zircons found *in situ* in thin section.

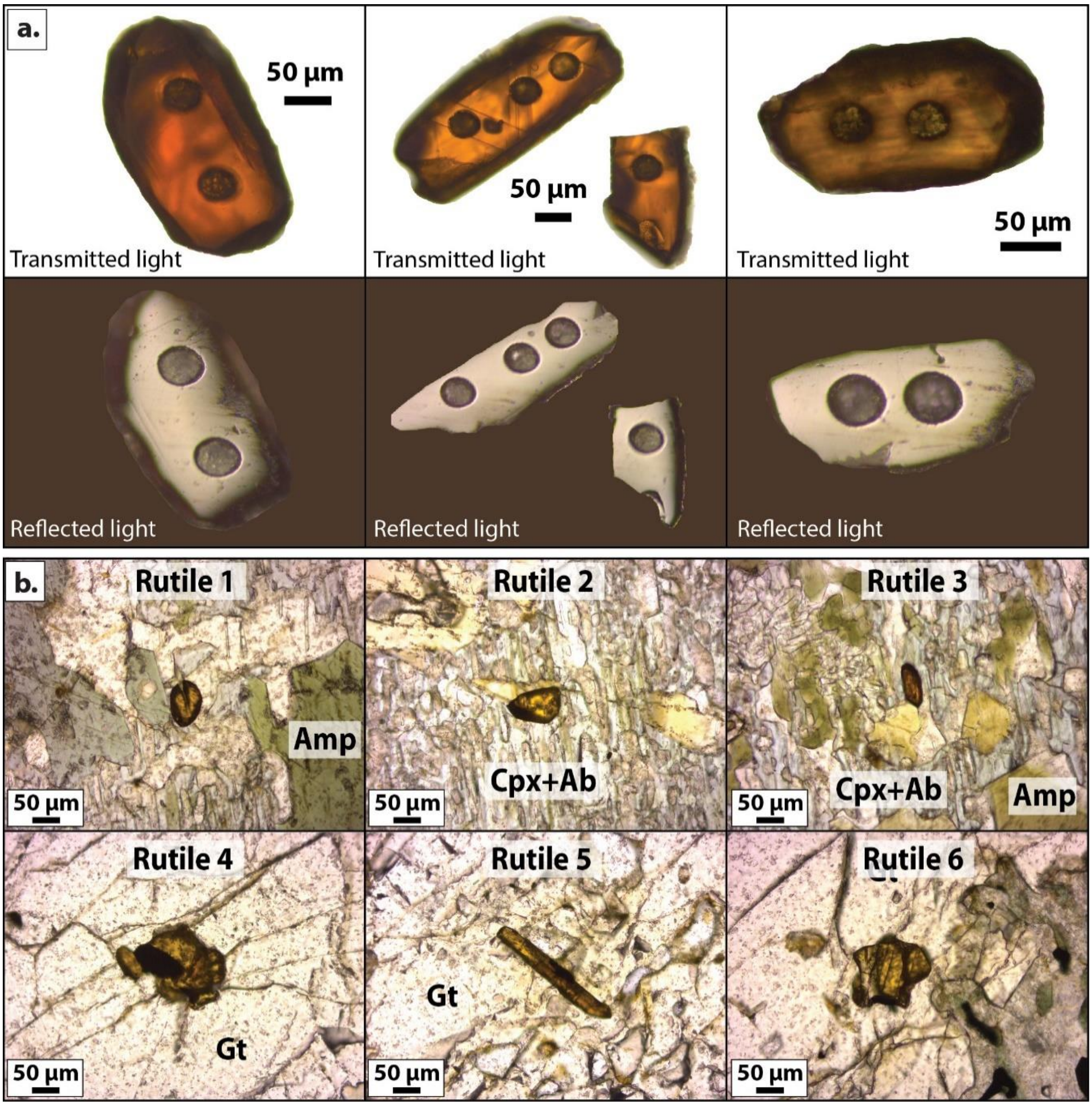

Fig.S7. Images of some studied rutiles for U-Pb dating. (a) Transmitted and reflected light images of sorted rutiles. (b) Microphotographs for six rutiles found *in situ* in thin section.

Supplementary Tables

Table S1. Chemical composition (in wt.%) of some selected main minerals in the eclogite obtained by Electron Microprobe Cameca SX-Five (Camparis, France). The omphacite compositions were estimated by the adding of the composition of clinopyroxene and the composition of albite-rich plagioclase measured in the matrix.

GARNET	Grt 59	Grt 60	Grt 61	Grt 62	Grt 63
SiO2	38,79	38,80	39,18	39,26	39,48
TiO2	0,10	0,06	0,10	0,07	0,01
Al2O3	20,45	20,14	20,06	20,63	21,06
FeO	26,76	27,43	27,43	26,93	26,33
MnO	2,72	2,95	2,36	1,67	1,03
MgO	2,98	2,96	3,29	3,77	4,10
CaO	9,13	8,77	8,62	8,43	9,21
Na2O	0,02	*bdl*	0,04	0,03	0,04
K2O	*bdl*	*bdl*	0,01	*bdl*	0,00
Cr2O3	0,02	0,02	0,02	0,02	0,01
Cl	0,01	0,01	0,01	*bdl*	0,02
**Total**	**100,98**	**101,15**	**101,11**	**100,82**	**101,30**
XGrossular	24,98	23,47	23,56	23,41	25,39
XPyrope	11,46	11,36	12,55	14,59	15,73
XAlmandine	57,39	58,06	58,69	58,30	56,62
XSpessartine	5,94	6,43	5,11	3,67	2,24

AMPHIBOLE	Amp 4	Amp 7	Amp 48	Amp 71	Amp 86
SiO2	42,69	46,92	45,21	48,77	45,88
TiO2	1,65	0,90	0,82	1,87	1,44
Al2O3	12,56	8,01	9,19	7,01	8,93
FeO	17,14	16,72	18,88	13,75	15,70
MnO	0,34	0,22	0,17	0,16	0,09
MgO	10,42	12,41	10,84	13,74	12,48
CaO	11,19	11,39	10,88	11,56	11,24
Na2O	2,18	1,51	1,74	1,19	1,66
K2O	0,17	0,10	0,14	0,18	0,20
Cr2O3	0,03	0,00	0,03	0,05	0,04
Cl	0,17	0,12	0,18	0,11	0,16
**Total**	**98,54**	**98,31**	**98,08**	**98,40**	**97,83**
Na+K	0,66	0,45	0,53	0,37	0,51
Si	6,35	6,91	6,75	7,07	6,79
XFe	0,48	0,43	0,49	0,36	0,41
XMg	0,52	0,57	0,51	0,64	0,59

CLINOPYROXENE	Cpx 53	Cpx 56	Cpx 77	Cpx 80	Cpx 81
**SiO2**	53,70	52,73	53,57	54,16	53,27
**TiO2**	0,06	0,02	0,23	0,14	0,23
**Al2O3**	1,02	1,46	1,47	1,97	2,37
**FeO**	10,85	11,33	8,85	9,06	9,18
**MnO**	0,24	0,22	0,02	0,05	0,11
**MgO**	12,64	12,47	13,26	13,36	12,87
**CaO**	22,22	21,67	22,24	21,57	21,89
**Na2O**	0,40	0,42	0,46	0,56	0,56
**K2O**	*bdl*	0,01	0,00	*bdl*	0,00
**Cr2O3**	0,05	*bdl*	0,02	*bdl*	0,03
**Cl**	0,00	0,01	0,00	*bdl*	0,00
**Total**	**101,19**	**100,35**	**100,13**	**100,87**	**100,51**
**xCa**	0,904	0,928	0,890	0,908	0,911
**xMg**	0,064	0,054	0,080	0,067	0,065
**xFe**	0,032	0,018	0,030	0,025	0,024
**xAl**	0,004	0,009	0,015	0,007	0,008
**xMg**	0,996	0,991	0,985	0,993	0,992
**xFe**	0,000	0,000	0,000	0,000	0,000
**Wollastonite**	0,595	0,588	0,595	0,582	0,591
**Enstatite**	0,042	0,049	0,044	0,060	0,048
**Ferrosilite**	0,363	0,363	0,360	0,358	0,361
**Ca-Mg-Fe Px**	0,620	0,620	0,618	0,615	0,615
**Jadeite**	0,022	0,010	0,025	0,030	0,030
**Acmite**	0,358	0,370	0,357	0,355	0,355

FELDSPAR	Pl 27	Pl 52	Pl 73	Pl 78	Pl 89
**SiO2**	64,46	65,68	63,65	61,73	62,28
**TiO2**	*bdl*	0,01	*bdl*	*bdl*	0,00
**Al2O3**	22,83	22,02	23,88	24,59	23,88
**FeO**	0,37	0,19	0,22	0,25	0,31
**MnO**	*bdl*	0,00	*bdl*	*bdl*	0,01
**MgO**	0,02	0,00	0,02	0,01	0,01
**CaO**	3,44	4,02	5,22	6,19	6,06
**Na2O**	9,05	9,66	8,63	8,23	8,40
**K2O**	0,52	0,13	0,11	0,12	0,03
**Cr2O3**	0,03	0,03	0,01	*bdl*	0,00
**Cl**	0,06	0,01	0,00	0,02	*bdl*
**Total**	**100,77**	**101,76**	**101,75**	**101,12**	**101,00**
**Xor**	3,03	0,70	0,64	0,66	0,17
**Xab**	80,15	80,76	74,48	70,20	71,39
**Xan**	16,82	18,54	24,88	29,14	28,45

OMPHACITE*	Cpx 77+Pl 78	Pl 52+Cpx 53
**SiO2**	57,65	59,69
**TiO2**	0,11	0,04
**Al2O3**	13,03	11,52
**FeO**	4,55	5,52
**MnO**	*bdl*	0,12
**MgO**	6,63	6,32
**CaO**	14,21	13,12
**Na2O**	4,35	5,03
**K2O**	0,06	0,06
**Cr2O3**	0,01	0,04
**Cl**	0,01	0,01
**Total**	**100,63**	**101,47**
**xCa**	0,793	0,781
**xMg**	0,151	0,148
**xFe**	0,056	0,070
**xAl**	0,002	*bdl*
**xMg**	0,998	1,012
**xFe**	0,000	0,000
**Wollastonite**	0,532	0,526
**Enstatite**	0,117	0,115
**Ferrosilite**	0,351	0,358
**Ca-Mg-Fe Px**	0,480	0,454
**Jadeite**	0,243	0,284
**Acmite**	0,277	0,262

RUTILE	Rt 38	Rt 39	Rt 40	Rt 69	Rt 91
**SiO2**	0,08	0,02	0,08	0,04	0,06
**TiO2**	101,18	101,27	100,46	100,84	100,19
**Al2O3**	*bdl*	*bdl*	*bdl*	*bdl*	*bdl*
**FeO**	0,30	0,33	0,33	0,28	0,67
**MnO**	*bdl*	*bdl*	*bdl*	0,02	0,04
**MgO**	0,00	*bdl*	*bdl*	*bdl*	*bdl*
**CaO**	0,00	0,06	0,07	0,09	0,13
**Na2O**	0,02	*bdl*	0,00	*bdl*	0,04
**K2O**	*bdl*	0,01	0,02	*bdl*	0,02
**Cr2O3**	0,04	0,00	0,06	0,08	0,01
**Cl**	0,01	*bdl*	*bdl*	*bdl*	*bdl*
**Total**	**101,63**	**101,69**	**101,02**	**101,34**	**101,15**

bdl: below detection limit

** estimated*

Table S2. Sm-Nd isotopic parameters for the eclogite sample obtained by HR-MC-ICP-MS Nu Plasma 1 (ULB, Belgium).

	**Sm (ppm)**	**Nd (ppm)**	**$^{147}Sm/^{144}Nd$**	**$^{143}Nd/^{144}Nd$ measured**	**2σ**	**$^{143}Nd/^{144}Nd$ at 2.216 Ga**	**ε ^{143}Nd at 2.09 Ga**	**ε $^{143}Nd_i$**
**RG-45977**	2,50	9,90	0,1525	0,512099	8	0,510001	+1,34	+2,04

Supplementary references

1- Delhal, J. *Le socle de la région de Luiza (Kasai)*. Musée royal de l'Afrique Centrale (1963).

2- Kabengele, M., Lubala, R. T., & Cabanis, B. Caractérisation pétrologique et géochimique du magmatisme ubendien du secteur de Pepa-Lubumba, sur le plateau des Marungu (Nord-Est du Shaba, Zaire). Signification géodynamique dans l'évolution de la chaîne ubendienne. *J. Afr. Earth Sci. (and the Middle East)* **13**(2), 243–265 (1991).

3- Lepersonne, J. *Notice explicative de la carte geologique du Zaire au 1/2 000 000*. Republique du Zaire, Department des Mines, Direction de la Geologie (1974).

4- Delhal, J., Ledent, D., & Torquato, J. R. Nouvelles données géochronologiques relatives au complexe gabbro-noritique et charnockitique du bouclier du Kasai et à son prolongement en Angola. *Ann. Soc. Géol. Belgique* (1976).

5- Delhal, J. Situation géochronologique 1990 du Précambrien du Sud-Kasai et de l'Ouest-Shaba. *Rapport Annuel Du Musée Royal de l'Afrique Centrale, Tervuren (Belgique), Département de Géologie et de Minéralogie*, 119–125 (1991).

6- Fieremans, C. The Luizian Trough in Zaïre and Angola and its relations with the Kasai Archaean complexes. *In:* Archaean cratonic rocks of Kasai and their influence on the evolution of the early Proterozoic. (B.T. Rumvegeri and F. Walraven, Eds.). *Newsletter IGCP Project* **273***(*1), 13-21 (1991).

7- Delhal, J., Lepersonne, J., & Raucq, P. *Le complexe sédimentaire et volcanique de la Lulua*. Musée royal de l'Afrique centrale (1966).

8- McDonough, W. F., & Sun, S.-S. The composition of the Earth. *Chem. Geol.* **120**(3-4), 223–253 (1995).

9- Kretz, R. Symbols for rock-forming minerals. *Am. Mineral.* **68**(1-2), 277–279 (1983).
